# TOBFAC: the database of tobacco transcription factors

**DOI:** 10.1186/1471-2105-9-53

**Published:** 2008-01-25

**Authors:** Paul J Rushton, Marta T Bokowiec, Thomas W Laudeman, Jennifer F Brannock, Xianfeng Chen, Michael P Timko

**Affiliations:** 1 Department of Biology, University of Virginia, Charlottesville, VA 22904, USA; 2 Academic Computing Health Sciences, Information Technology and Communication, University of Virginia, Charlottesville, VA 22908, USA; 3 Department of Microbiology, University of Virginia, Charlottesville, VA 22908, USA

## Abstract

**Background:**

Regulation of gene expression at the level of transcription is a major control point in many biological processes. Transcription factors (TFs) can activate and/or repress the transcriptional rate of target genes and vascular plant genomes devote approximately 7% of their coding capacity to TFs. Global analysis of TFs has only been performed for three complete higher plant genomes – Arabidopsis (*Arabidopsis thaliana*), poplar (*Populus trichocarpa*) and rice (*Oryza sativa*). Presently, no large-scale analysis of TFs has been made from a member of the *Solanaceae*, one of the most important families of vascular plants. To fill this void, we have analysed tobacco (*Nicotiana tabacum*) TFs using a dataset of 1,159,022 gene-space sequence reads (GSRs) obtained by methylation filtering of the tobacco genome. An analytical pipeline was developed to isolate TF sequences from the GSR data set. This involved multiple (typically 10–15) independent searches with different versions of the TF family-defining domain(s) (normally the DNA-binding domain) followed by assembly into contigs and verification. Our analysis revealed that tobacco contains a minimum of 2,513 TFs representing all of the 64 well-characterised plant TF families. The number of TFs in tobacco is higher than previously reported for Arabidopsis and rice.

**Results:**

TOBFAC: the database of tobacco transcription factors, is an integrative database that provides a portal to sequence and phylogeny data for the identified TFs, together with a large quantity of other data concerning TFs in tobacco. The database contains an individual page dedicated to each of the 64 TF families. These contain background information, domain architecture via Pfam links, a list of all sequences and an assessment of the minimum number of TFs in this family in tobacco. Downloadable phylogenetic trees of the major families are provided along with detailed information on the bioinformatic pipeline that was used to find all family members. TOBFAC also contains EST data, a list of published tobacco TFs and a list of papers concerning tobacco TFs. The sequences and annotation data are stored in relational tables using a PostgrelSQL relational database management system. The data processing and analysis pipelines used the Perl programming language. The web interface was implemented in JavaScript and Perl CGI running on an Apache web server. The computationally intensive data processing and analysis pipelines were run on an Apple XServe cluster with more than 20 nodes.

**Conclusion:**

TOBFAC is an expandable knowledgebase of tobacco TFs with data currently available for over 2,513 TFs from 64 gene families. TOBFAC integrates available sequence information, phylogenetic analysis, and EST data with published reports on tobacco TF function. The database provides a major resource for the study of gene expression in tobacco and the *Solanaceae *and helps to fill a current gap in studies of TF families across the plant kingdom. TOBFAC is publicly accessible at .

## Background

Tobacco [*Nicotiana tabacum L*.] is a member of the agriculturally important *Solanaceae *and is one of the most studied higher plant species. This is because of both its economic importance and because it is a convenient plant system for research. Tobacco can be easily transformed and has a relatively short generation time. A system of reduced complexity, the tobacco Bright Yellow-2 (BY-2) cell line, is also available and this cell line is fast growing, responds to a variety of plant hormones and can be stably transformed [1]. BY-2 cells are an excellent experimental system for studies of gene expression and secondary metabolism. The one missing piece in the puzzle is the availability of the genome sequence of tobacco.

The large genome size of tobacco (approximately 4.5 Gb) makes the goal of sequencing the tobacco genome difficult. Fortunately, there are now a number of methods that can deliver sequence information on the vast majority of genes in a species without the need to sequence and assemble the entire genome. One of these techniques is methylation filtration (MF), which preferentially clones the hypomethylated fraction of the genome, effectively reducing the size of the genome to be sequenced. MF has already been successfully applied in maize, sorghum and cowpea [2-5]. The development of MF followed studies of genome architecture that revealed that repetitive elements tend to form clusters within plant genomes that become heavily methylated (hypermethylated), leaving stretches of less-methylated (hypomethylated), low-copy gene-rich space scattered in islands throughout the genome [6,7].

The Tobacco Genome Initiative (TGI) has obtained sequence from an estimated minimum of 90% of tobacco gene space (cultivar Hicks Broadleaf) using MF technology [8]. We have used a dataset of 1,159,022 gene-space sequence reads (GSRs) generated by the TGI as the basis for identifying the majority of all members of 64 well-characterized transcription factor (TF) families. Our dataset is estimated to represent a minimum of 2,513 genes and TOBFAC has been designed not only to be a repository for these sequences but also to be a major resource for all data concerning tobacco TFs.

Since the publication of the first version of the Arabidopsis genome sequence, finding the complete set of known TFs in plant genomes has become an attainable goal [9]. However, only three higher plant genome sequences are currently available, Arabidopsis (*Arabidopsis thaliana*), poplar (*Populus trichocarpa*) and rice (*Oryza sativa *L.). A number of databases have been constructed that integrate all predicted TFs from these three genomes. These include the Plant Transcription Factors databases [10,11], PlnTFDB [12] and PlanTAPDB [13]. In addition to these, there are a number of similar databases devoted to other species, but these are based on EST data. The species contained in these EST-based datasets include maize, wheat, barley, sorghum, sugarcane, cotton soybean, potato, tomato, apple, orange, grape, sunflower, lotus, loblolly pine and white spruce [14]. However, none of these are devoted to tobacco and none approach the level of coverage and total of over 2,500 TFs that we have documented from tobacco. The *Nicotiana tabacum *genome is the result of an interspecific hybridization event between *Nicotiana sylvestris *and *Nicotiana tomentosiformis *resulting in an allopolyploid genome with a basic chromosome number of x = 24. Based on a high density microsatellite-based genetic map, there is clear evidence of several large translocation events between the ancestral chromosomes but no evidence of large duplication events. The extent of loss of chromosomal segments is difficult to assess in the absence of diploid maps for the ancestral species. It is therefore difficult to predict the total number of tobacco TFs, but it is likely to be over 3,000. After the databases that cover Arabidopsis, rice and poplar, TOBFAC is currently the most extensive species-specific higher plant TF database. It will be a major resource for the study of gene expression in tobacco and the *Solanaceae *and will also facilitate studies of TF families across the plant kingdom.

## Construction and Content

### Source dataset and knowledgebase construction

A total of 1,159,022 GSRs obtained by methylation filtering were downloaded from the TGI (March 6^th ^2006) [8]. The GSRs were imported into a PostgrelSQL database having a schema very similar to that used for the Cowpea Genomics Initiative [5]. Scripting was performed in Perl using Perl DBI/DBD for PostgrelSQL database connectivity [15]. The Perl modules HTML::Template and HTML::FillInForm were used to facilitate the a Model-View-Controller software architecture which separates the display layer (HTML) from bioinformatics logic layer (Perl code). In addition to sequence data, the database stores Blast results as well as some ancillary meta data. The TOBFAC web pages are conceptual views of the PostgrelSQL relational database. Some web pages combine records from multiple tables, and link to static files outside the database.

### Identification and classification of tobacco TFs

**Figure 1:**
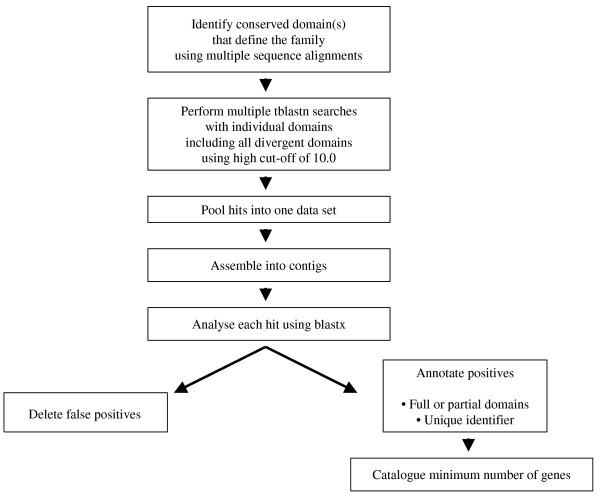
Pipeline for the identification of tobacco TF sequences from the 1,159,022 GSRs.

**Figure 2:**
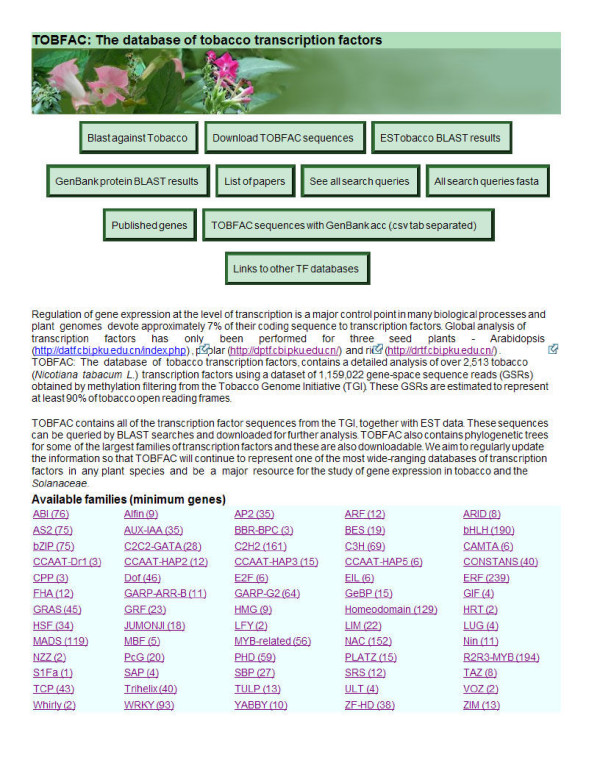
Visualization of part of the TOBFAC web interface. Links at the top of the main page enable downloading of both the complete set of TOBFAC sequences and the complete set of sequences that were used in the searches to find them. There are also links to the homology search page, the published tobacco TF page, a list of publications on tobacco TFs, BLAST results against the Genbank protein database and links to other TF databases. The list of available families links to an individual page for each of the 64 analysed TF gene families.

**Figure 3:**
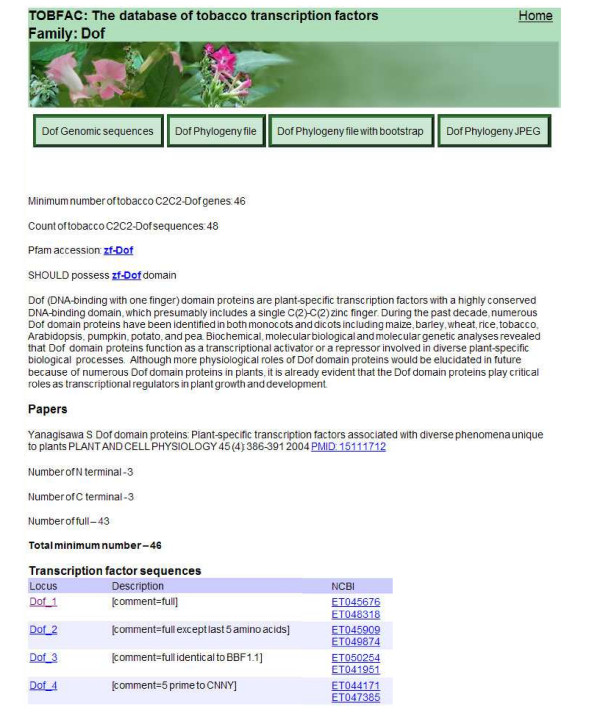
Snapshot of a TF family page. These contain all the sequences for each tobacco TF family both individually and as a single file of the complete family. Each predicted gene is listed individually, together with all of its' component GSRs. The GSRs are presented as hotlinks to their Genbank entries. The page also contains a short description of the family with links to the Pfam domains, downloadable phylogenies and JPEGs of the phylognetic trees, and the sequences that were used to search the GSRs to find members of this family. The total number of sequences and minimum number of tobacco genes in this family are shown at the top of the page.

To isolate GSRs encoding tobacco TFs, multiple independent tblastn searches of the GSR dataset were performed using different versions of the amino acid sequences of the conserved domains from each of 64 TF families. In most cases, the conserved sequences for each gene family were taken from The Database of Arabidopsis Transcription Factors (DATF) [11]. Where the conserved sequences were taken from other sources, this is noted. The exact amino acid sequences used for the searches are listed on the TOBFAC website, both for individual gene families and as a complete list that can be downloaded to facilitate similar searches of other datasets. Our aim was to isolate every gene in all TF gene families and we therefore performed at least 5–10 independent searches for each gene family. In particular, all TF family members that showed divergence from the consensus sequence were used in searches so that divergent or novel members were found. We combined this multiple search strategy with a cut off e-value of 10 in order to rigorously ensure that all possible gene family members were found (false positives were later discarded). For each TF family, the GSR hits from all searches were combined into one data set and assembled into contigs using a local web based implementation of the Phrap program for contig building that is available on the Cowpea Genespace/Genomics Knowledge Base (CGKB) [5]. Each contig/singleton was then individually analysed by BLASTX searches against the non-redundant protein database housed at NCBI [16]. Any sequences that did not contain the conserved domain for the TF family under analysis were discarded at this point (see Figure 1). The sequences that remained represented *bona fide *tobacco TFs and each was given a unique identifier. These sequences were uploaded to TOBFAC, where the complete list of tobacco TF sequences is available as one dataset (see Figure 2). For each TF gene family, the page dedicated to the family has each gene listed individually, together with the accession numbers of the GSRs that form that predicted gene (see Figure 3). In some cases, predicted genes were found to correspond to previously published genes encoding tobacco TFs, and where this is the case, their published name is listed as an alias.

### Determination of the minimum number of genes in each TF family

It is estimated that at least 90% of tobacco TF genes have been tagged by the MF technology [8], but not all of these genes are present as complete gene sequences. A major task was therefore to calculate the minimum number of genes present in each tobacco TF family based on the GSRs. This was calculated based on the number of independent sequences that contained a certain portion of the conserved domain (say amino acids 20–30). The largest number of copies of any part of the domain represents the minimum number of those genes in tobacco. For larger gene families, the genes were first assigned to known subfamilies and the calculation was performed for each subfamily. The final number for the gene family was then the sum of all the subfamilies.

### Phylogenetic analysis of TF families

**Figure 4:**
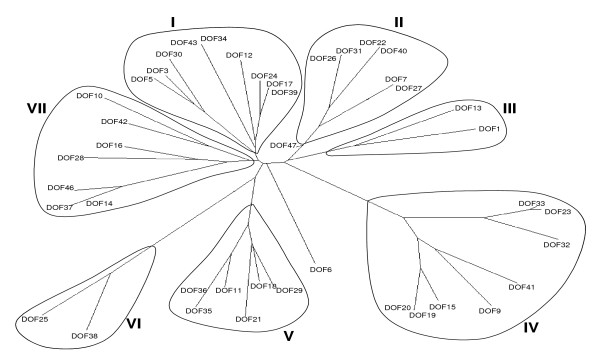
Phylogenetic analysis of Dof genes. Phylogenetic and molecular evolutionary analyses were conducted using MEGA version 4. Phylogenetic trees were produced using the amino acid sequences of the Dof domains by the Neighbor-Joining method. The data are presented as an unrooted tree to provide an overview of the gene family.

Phylogenetic and molecular evolutionary analyses were conducted using MEGA version 4 [17]. Phylogenetic trees were produced by the Neighbor-Joining method and statistical support for the nodes in the phylogenetic trees (bootstrap values from a minimum of 1000 trials) were obtained for each tree. TOBFAC users can download the phylogenetic tree files, either with or without bootstrap values, and display them in any desired format. Currently, phylogenetic analysis is only available for some of the major TF gene families (ERF, WRKY, NAC, homeodomain, bZIP, bHLH, R2R3MYB, MADS box, SAP, BES and Dof genes) (see Figure 4). As additional phylogenetic information is generated for other TF families this will be made available on TOBFAC.

### Comparison of TOBFAC sequences and EST sequences

**Figure 5:**
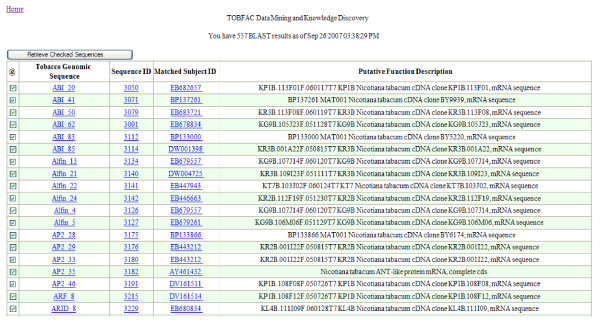
Snapshot of the page showing the results of BLAST searches with the tobacco TF contigs against 46,546 tobacco EST sequences from the ESTobacco project [18]. A total of 557 of the transcription factor contigs are supported by EST sequences.

46,546 tobacco EST sequences from the European Sequencing of Tobacco Project [18] were uploaded to TOBFAC. BLAST searches were performed with the TOBFAC TFs against the EST dataset. A total of 557 of the TFs are supported by EST sequences (see Figure 5). This is less than 20% of the total and shows that the GSRs have achieved a much greater coverage than the ESTs. This low level of EST support is probably because some TFs are very tissue and developmental stage specific with brief and low levels of expression. Additional EST datasets from tobacco will be subject to similar analysis as they become available. Combined EST and GSR datasets will, in the future, result both in a more robust dataset (due to the linking of partial sequences into single gene sequences) and a slight increase in the minimum number of predicted TFs in tobacco.

## Utility

### The main page for TOBFAC

TOBFAC is one of the most extensive species-specific higher plant TF databases, with only Arabidopsis, rice and poplar having more complete TF analysis to date [13]. The data will provide a major resource for the study of gene expression in tobacco and the *Solanaceae *and will also facilitate studies of TF families across the plant kingdom. The current version will be updated regularly to improve the dataset of tobacco TFs. The main page for TOBFAC is the main web interface for all information contained in the database (see Figure 2). It contains introductory information about both tobacco and the GSR project conducted by the TGI. Links at the top of the main page enable downloading of both the complete set of TOBFAC sequences and the complete set of sequences that were used in the searches to find them. It is also possible to download the complete list of predicted tobacco genes and the Genbank entries for their individual constituent GSRs. There are also links to the homology search page, the published tobacco TF page and the list of publications on tobacco TFs. The published tobacco TF page contains a list of published tobacco TF genes sorted by gene family and with hotlinks to both Genbank and PubMed entries. The list of publications on tobacco TFs is reached via a hotlink on the main page and contains publications again sorted by gene family and with hotlinks to PubMed entries and/or PDF files for all publications. A list of all 64 available TF gene families is present on the main page together with the minimum number of tobacco genes in each family. The TF family names link to the individual TF family pages.

### The individual TF family pages

Each TF family has its own dedicated page (see Figure 3) that contains all sequences from this group. A button at the top of the page links to the complete list of sequences in the gene family. Other buttons allow downloading of the phylogenetic tree file, either with or without bootstrap values, and a JPEG of the phylogenetic tree. A short introduction describes the family and there is also a link to the family's Pfam accession. All predicted sequences are presented, together with what parts of the gene they each contain. An explanation of how the minimum number of genes in this gene family was calculated from this total number of sequences is also present. Below the introduction, each individual gene is listed together with links to the Genbank entries for every individual GSR that makes up this gene. The gene name links to the individual page for the gene itself. There is also a list of papers that relate to this familt in tobacco, complete with links to their PubMed entries. At the bottom of each TF family page is a list of all published tobacco genes that belong to this family, together with links to the Genbank entries for these genes. This wealth of tobacco-related features should provide the user with most of the available information on this gene family in tobacco together with several different ways to utilise the TOBFAC sequences.

### The individual gene pages

Each TF family page lists all of the genes in that family in the TOBFAC database. Each gene name links to a page dedicated to that gene that contains a wealth of information designed to aid research into that gene. At the top of the page is the DNA sequence of the gene in fasta format followed by a six frame translation of the sequence. Below that is a link to the pfam accession page. If a gene has already been published, this is stated together with the name that it was published under and links to PubMed and Genbank entries for the gene. This enables integration of all available data on TOBFAC genes that have already been published. Additional data on the predicted gene follows with results from searches of the EST data and Genbank. The top hits are presented, together with their definition lines, and links to the database entries for the sequences. Below the searches are links to all Genbank entries of all of the individual GSRs that make up the predicted genes. Finally, the individual gene pages also contain the complete lists for the gene family of both relevant papers and published genes. Each individual gene page therefore integrates a large amount of data from multiple sources, contains multiple functionalities and provides a one-stop-shop environment for research into each of the 2,882 TOBFAC TF sequences.

### The homology search page

The homology search page is reached via a link on the main page and is a local installation of the NCBI BLAST program that allows searching against the TOBFAC sequences. It can also be used to search the 46,546 tobacco EST sequences from the ESTobacco project [18] or the tobacco unigenes from Genbank. The integration of these datasets facilitates a "one stop shop" approach to the study of tobacco TFs. We have also performed BLAST searches with the TOBFAC sequences against the ESTobacco EST sequences. A total of 557 of the TOBFAC tobacco TFs are supported by EST sequences (see Figure 5). A list of TOBFAC genes that are supported by ESTs is available via a link on the main page.

### The published tobacco TF page

TOBFAC aims to contain information on all known tobacco TF genes. This includes not only information from the TGI GSR project and EST sequences but also a list of published tobacco TF genes. The published tobacco TF page contains a list of published tobacco TF genes sorted by gene family and with hotlinks to both Genbank and PubMed entries. This list of genes facilitates the integration of data from multiple sources. Some care should be exercised, however, when comparing published sequences with GSR sequences because different ecotypes have been used in many cases and also published sequences have been obtained from the BY-2 cell line. For some of the larger gene families, we have tried to integrate published and GSR sequences, and although this has often been successful, this was not always the case. For this reason, future versions of the TOBFAC dataset will primarily be centered on GSR data and corresponding EST sequences.

### The list of publications on tobacco TFs

TOBFAC is designed not only to be a database of all known tobacco TF sequences, but also a knowledge base of all tobacco TF-related material. A list of publications on tobacco TFs is reached via a hotlink on the main page and contains publications sorted by gene family and with hotlinks to PubMed entries and/or PDF files. Over half of the tobacco TF families have no published literature concerning them and this illustrates how the information contained in TOBFAC will facilitate novel areas of research in tobacco.

## Discussion and Conclusion

Genome-related public databases are invaluable to the scientific community and transcriptional regulation of gene expression is a major control point in many biological processes. Databases that contain information on complete or near-complete complements of TFs are major resource for the plant research community but this does not currently include a member of the *Solanaceae*, one of the most important families of vascular plants. TOBFAC fills this void and contains the majority of tobacco TFs (a minimum of 2,513) from 64 gene families together with extensive data on each gene family. TOBFAC integrates these TF sequences with published tobacco TFs, scientific literature on tobacco TFs, phylogenetic trees and EST data. The aim is to make TOBFAC the database of choice for transcription-related projects in tobacco and an important resource for projects in other members of the *Solanaceae*.

The availability of all, or nearly all, members of TF families from several species will facilitate the study of their biological functions, phylogenetic relationships, and the evolution of their DNA-binding domains [12]. The dataset contained within TOBFAC has already been used for phylogenetic analysis of the ERF, WRKY, NAC, homeodomain, bZIP, bHLH, R2R3MYB, CONSTANS, ZIM, Dof and MADS box genes. These phylogeneies have been successfully used to both predict gene function and to find novel subfamilies of TF genes (Rushton, P.J., manuscript in preparation).

Gene space sequences have been published for maize, sorghum and cowpea [2,3,5] and the near future will see this number increase. The computational pipeline described in this article (see Figure 1) can be widely applied to any new GSR dataset and modified to include any new families of TFs as they are discovered. We believe it to be a robust method that increases the chances of isolating TF gene sequences from GSR datasets, especially for short or incomplete sequences and divergent members of gene families. This is in contrast to other approaches that use stringent filtering processes to avoid false-positives but can thereby fail to identify up to 20% of TF genes [13].

### Future plans and releases

The current version of TOBFAC is based on 1,159,022 GSRs obtained from the TGI website. As additional GSRs, ESTs and BAC sequences become available, we will integrate these new sequences into TOBFAC and update both TF family data and phylogenetic analyses. Microarray-based transcription profiling is currently being performed for tobacco and expression data for TFs will also be included in future versions of TOBFAC.

## Availability and requirements

Project name: TOBFAC: The database of tobacco transcription factors.

Project home page: .

Operating system: Platform independent.

Programming language: Perl.

Other requirements: None.

Licence: None required.

Any restrictions to use by non-academics: None.

## Authors' contributions

PJR designed the project, performed data analysis and assisted in the design of the database. MTB performed data analysis, assisted in the design of the database, and performed the analysis of published papers and genes. TWL participated in the design of the database and database system administration. JFB performed data analysis. XC performed the bioinformatics data analysis and web implementation. MPT served as the principal investigator of the project. All authors have assisted in writing of the manuscript and have read and approved the final submitted version. PJR and MTB are equal first authors.
